# Prevalence of Sexually Transmitted Infections among Married Women in Rural Nepal

**DOI:** 10.1155/2018/4980396

**Published:** 2018-08-26

**Authors:** Sunila Shakya, Solveig Thingulstad, Unni Syversen, Svein Arne Nordbø, Surendra Madhup, Krista Vaidya, Biraj Man Karmacharya, Bjørn Olav Åsvold, Jan Egil Afset

**Affiliations:** ^1^Department of Clinical and Molecular Medicine, Faculty of Medicine and Health Sciences, Norwegian University of Science and Technology, Trondheim, Norway; ^2^Department of Gynecology and Obstetrics, Dhulikhel Hospital/Kathmandu University School of Medical Sciences, Kavre, Nepal; ^3^Department of Obstetrics and Gynecology, St Olav's Hospital, Trondheim, Norway; ^4^Department of Endocrinology, St Olav's Hospital, Trondheim, Norway; ^5^Department of Medical Microbiology, St Olav's Hospital, Trondheim, Norway; ^6^Department of Microbiology, Dhulikhel Hospital/Kathmandu University School of Medical Sciences, Kavre, Nepal; ^7^Department of Public Health and General Practice, Faculty of Medicine, Norwegian University of Science and Technology, Trondheim, Norway; ^8^Department of Community Medicine, Dhulikhel Hospital/Kathmandu University School of Medical Sciences, Kavre, Nepal

## Abstract

**Introduction:**

We have previously determined the prevalence of human papillomavirus (HPV) infection among women in rural Nepal. In the current study, we also wanted to examine the prevalence of and risk factors for other sexually transmitted infections (STIs) in the same population.

**Methods:**

Population-based study of nonpregnant women ≥ 15 years who were married or had a history of marriage in the past, residing in five rural villages in Nepal. Data on sociodemographic characteristics, reproductive history, and genitourinary symptoms were collected, and a gynecological examination was conducted. Cervical samples were analyzed by real-time PCR for* Neisseria gonorrhoeae*,* Chlamydia trachomatis*, and* Trichomonas vaginalis* and HPV, and a serum sample was analyzed for syphilis, hepatitis B virus (HBV) and HIV infection by serology.

**Results:**

Of 2416 eligible women, 62% participated. Trichomoniasis,* Chlamydia trachomatis* infection, HPV and HBV infection, and syphilis were detected in 5.4%, 0.8%, 14.3%, 0.3%, and 0.2% of the women. None had gonorrhea or HIV infection. Of those with genitourinary symptoms, 6.3% had a curable STI. Vaginal discharge classified as abnormal by gynecological examination, but not self-reported discharge, was significantly associated with laboratory diagnosis of a curable STI. Risk factors for trichomoniasis were reproductive age and high cast/ethnicity. Due to low prevalence, risk factors for other STIs could not be disclosed.

**Conclusion:**

We observed high prevalence of HPV infection followed by trichomoniasis, while other STIs were rare among women in rural Nepal. There was no association between genitourinary symptoms and laboratory-confirmed STIs.

## 1. Introduction

Sexually transmitted infections (STIs) cause considerable morbidity worldwide, especially among women of reproductive age. All STIs are preventable, while only gonorrhea (*Neisseria gonorrhoeae*),* Chlamydia trachomatis* infection, syphilis, and trichomoniasis (*Trichomonas vaginalis*) are curable [1, 2]. Viral infections caused by hepatitis B virus (HBV), human immunodeficiency virus (HIV), human papillomavirus (HPV), and herpes simplex virus (HSV) are currently noncurable [2].

According to estimates by the WHO, nearly 360 million new cases are infected with the four curable STIs every year [2]. Among these, 78.5 million occur in Southeast Asia. Data on STI prevalence in Nepal are limited, and prevention and control programs have mainly focused on HIV infection. According to national reports, the estimated prevalence of HIV infection was 0.2 - 0.3% in 2013 [3, 4]. In high-risk populations, however, there has been a concentrated HIV epidemic with a prevalence of 1.7 - 18.3% [3, 5, 6]. So far, data on other STIs from Nepal are mostly hospital- or health-camp based or from high-risk populations [5–9].

Nepalese national guidelines on “Case Management of Sexually Transmitted Infections” recommend a syndromic approach with or without the use of laboratory tests, depending on locally available resources, as recommended by the WHO [10, 11]. Due to lack of laboratory facilities or unavailability of tests, most STI diagnoses in Nepal are based on this approach, both in rural and most of the urban areas. An exception to this practice is patients with a high suspicion of HIV infection who are advised to go for laboratory confirmation. At the rural setting, STI diagnoses are frequently based solely on presented symptoms, due to reluctance to undergo a vaginal examination or lack of gynecological equipment at the health facility [11]. Women are often unable to distinguish between physiological and abnormal vaginal discharge [12]. Since symptoms from the genitourinary tract are rarely caused by a STI, a syndrome-based therapy may result in overtreatment of those with vaginal discharge. Another concern with the syndromic approach is that asymptomatic infections, which represent a major proportion of STIs, are not detected [13]. Until simple and affordable laboratory tests for diagnosis of STIs become widely available, population-based prevalence data are important to optimize syndromic-based STI management protocols.

We have previously investigated the prevalence of HPV among women in rural Nepal [14]. In the present study, we aimed to examine the prevalence of HPV and other STIs in the same population for comparison, namely, the curable STIs gonorrhea,* Chlamydia trachomatis* infection, syphilis, and trichomoniasis, as well as viral STIs caused by HBV, HIV, and HSV. Furthermore, we wanted to explore risk factors for these STIs.

## 2. Materials and Methods

### 2.1. Study Site and Study Population

The study was conducted in the period from February 2012 to May 2013 in Kavre district in the hilly region of Nepal. The study site and population have been described in our previous study focusing on HPV infection [14]. Briefly, all nonpregnant women ≥ 15 years of age from five villages who were or had been married were invited to a one-day screening. Women who had never been married and pregnant women were not recruited, since they according to local tradition should not undergo gynecological examination. Written informed consent was obtained from those who agreed to participate in the study.

### 2.2. Data Collection and Questionnaire

Seven trained female interviewers (auxiliary nurse midwives and a public health officer) administered a questionnaire. Information on the women's sociodemographic characteristics, reproductive history, and genitourinary symptoms was obtained. Cast/ethnicity of the participants was broadly categorized according to their ancestry, language, culture, and tradition. Participants who had attended a public or private school, irrespective of number of years completed, were categorized as having formal education.

### 2.3. Clinical Examination and Specimen Collection

A gynecological examination was performed by either a gynecologist (Sunila Shakya) or by an experienced nurse and findings were noted. Two cervical samples were obtained from each participant; one was obtained using a cyto-brush (Rovers® Cervex-Brush® Combi, Norway) which was transferred to Copan Universal Transport Medium (UTM-RT, Copan Italia, Brescia, Italy), and the other was collected by Sigma Trans-swab (Medical Wire & Equipment, Wiltshire, UK). Blood samples were drawn in a VACUETTE® TUBE 4 ml Z Serum Separator Clot Activator (Greiner Bio One International, Frickenhausen, Germany). The samples were transported on ice to the laboratory at Dhulikhel Hospital/Kathmandu University School of Medical Sciences (DH/KUSMS) on the day of collection. The cervical samples were stored at -20°C until further analyses. Blood samples were centrifuged for 10 min at 2000 rpm as soon they reached the laboratory and then stored at -70°C until further analyses.

### 2.4. Culture of Cervical Specimens

Within 4 to 12 hours of sample collection, cervical specimens in the Sigma Trans-swabs were inoculated onto chocolate and blood agar and were incubated at 37°C for 72 hours for culture of* N. gonorrhoeae*.

### 2.5. DNA Extraction and PCR Analysis

DNA was extracted from cervical samples stored in UTM-RT medium according to the manufacturer's instructions. Extracted DNA was stored at -20°C until transported frozen to the Norwegian University of Science and Technology (NTNU)/ St. Olav's Hospital, Trondheim, Norway, for analysis. Detection of* N. gonorrhoeae*,* C. trachomatis,* and* T. vaginalis* was done by in-house real-time PCR with primers and probes as described previously [15, 16]. HPV DNA detection was done using the Anyplex™II HPV28 Detection real-time multiplex polymerase chain reaction (PCR) assay (Seegene Inc., Seoul, Korea) as described in Shakya et al. [14]. A detailed description of PCR methods used in the study is available as Supplementary Material (available here).

### 2.6. Serological Analyses

Primary serological tests were performed at the laboratory at DH/KUSMS, before positive sera were transported to NTNU/St. Olav's Hospital, Trondheim, Norway, for confirmation. The sera were tested for HBsAg by AccuDiag™ HBsAg Elisa (Diagnostic Automation/Cotez Diagnostics, Inc., CA, USA), and positive results were confirmed by VIDAS HBsAg Ultra neutralization test (bioMérieux, Marcy l'Etoile, France). The sera were tested for HIV by AccuDiag™ HIV Elisa (Diagnostic Automation/Cotez Diagnostics, Inc., CA, USA). Sera were initially assessed for syphilis by RPR (RFCL Diagnova Trepostat, Ranbaxy, Gurgaon, India), and reactive sera were confirmed by AccuDiag™ Syphilis IgG/IgM ELISA (Diagnostic Automation/Cotez Diagnostics), Serodia-TP-PA (Fujirebio Europe, N.V., Gent, Belgium), and Capita NMT Syphilis IgM EIA (Trinity Biotech plc, Wicklow, Ireland).

### 2.7. Statistical Analyses

Statistical analyses were done using SPSS software (IBM SPSS version 21). The prevalence of overall and individual STIs according to sociodemographic and reproductive characteristics is presented with 95% confidence intervals (CIs). The prevalence of curable STIs among women with and without symptoms is also given, and Chi-square (**χ**^2^) test was used to evaluate the difference between the two groups. Associations of sociodemographic and reproductive characteristics with trichomoniasis were estimated using logistic regression analysis, with adjustment for the potential confounders age, cast/ethnicity, participants' educational status, age at marriage, and participant's and husband's number of marriages.

### 2.8. Ethical Approval

The study was approved by the Nepal Health Research Council (Approval no. 124/2012), the DH/KUSMS Institutional Review Committee (Approval no. 38/11), and the Regional Committee for Medical and Health Research Ethics, Central Norway (REK no. 2011/2540)

## 3. Results

### 3.1. Study Participants and Sample Collection

**Figure 1:**
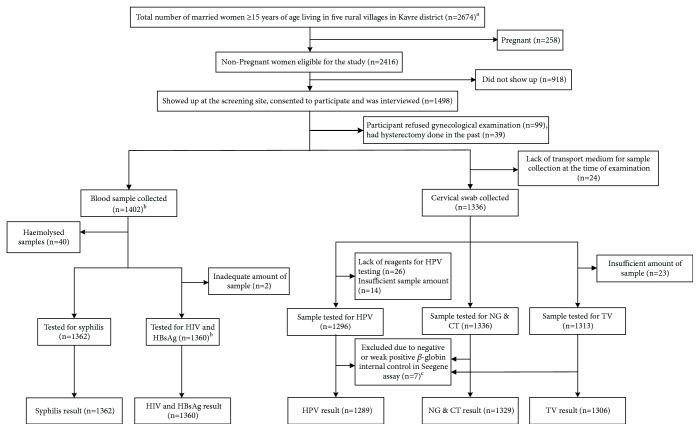
Flow chart showing selection of study participants, sample collection, and analyses. ^a^Villages: Bolde Fediche, Thulopersel, Pokhari-Naranyanthan, Saramthali, and Sarasyunkharka. ^b^Refused blood sample collection (n=96). ^c^Samples with negative or weakly positive *β*-globin gene internal control for human DNA in the Seegene Anyplex™II HPV28 real-time PCR were considered to have inadequate amount of sample or contained inhibitors and were therefore excluded from further analyses; CT,* Chlamydia trachomatis*; NG,* Neisseria gonorrhoeae*; HPV, human papillomavirus; TV,* Trichomonas vaginalis*.

In total, 1498 (62%) of 2416 eligible women were recruited and completed the questionnaire (Figure 1). Among those who did not participate in the study, 250 were pregnant, whereas data were lacking on the remaining nonparticipants. Sociodemographic characteristics are shown in Table 1. The median age was 40 (range 17-86) years. The majority of the women (83.8%) belonged to the Adhivasi/Janajati, a disadvantaged low cast. The mean age at marriage was 18 (range 5-38) years, but 24.7% of the participants were married before they turned 16. Among 1313 (91.9%) women who were still married, 79.1% were living with their husband, and 12.3% and 8.6% had husbands who were labor migrants within Nepal or overseas, respectively. The majority of the women (96.5%) and their husbands (88.7%) had been married only once (Table 1). Agriculture and/or livestock farming were the main income sources, but only one-third reported an own source of income. 64.9% of the women and 49% of the husbands were illiterate. The majority of the husbands (94.1%) had never used condom, and 92.9% of the women had never used an intrauterine contraceptive device.

A cervical sample could not be collected from 162 women (10.8%), either because of refusal to undergo a gynecological examination (n=99), hysterectomy (n=39), or lack of transport medium/containers at the study site for sample collection (n=24). Consequently, cervical samples from 1336 women were available for PCR analyses of* C. trachomatis* and* N. gonorrhea* and 1312 for PCR analysis of* T. vaginalis*. Due to inadequate sample quantity and/or lack of reagents, the number of cervical samples for various tests differed.

Blood samples were collected from 1402 women (96 women refused); 40 of these samples were hemolysed. Thus, test for syphilis was done on 1362 serum samples. Due to inadequate amount of serum, 1360 samples were available for HIV and HBsAg analyses. At least one sample, either a cervical or blood sample or both, was available from 1428 women.

### 3.2. Prevalence of STIs

In total, 250 women had at least one STI, including HPV, resulting in an overall prevalence of 17.5% (Table 2). Eighty-two (5.7%) of these had a curable STI. The prevalence of trichomoniasis and* C. trachomatis* infection was 5.4% and 0.8%, respectively. 14.3% had HPV infection. Seven samples tested initially positive for HBsAg, but only four of these were confirmed in the neutralization test, giving a prevalence of HBV infection of 0.3%. Three samples with a positive primary test result for syphilis had titers from 640->20 000 in the TPPA-test. One sample had a RPR-titer of >32 and positive IgM-test, indicating recent infection, while the two other samples had a titer of 1-2, with negative IgM-test, indicating later stage of disease or previous infection. Altogether, the prevalence of syphilis was 0.2%.* N. gonorrhea *was not detected in any of the women, neither by culture nor PCR analysis, and none were found to have HIV infection.

None of the women had genital lesions that gave suspicion of current HSV infection. Nine women had more than one STI; five had* C. trachomatis* and HPV infection, two had trichomoniasis,* C. trachomatis, *and HPV infection, one had trichomoniasis and HBV infection, and one woman had syphilis and coinfection with different HPV genotypes.

### 3.3. STI versus Reported Symptoms and Clinical Findings

One or more genitourinary symptoms were reported by 648 (45.4%) of the participants, like abnormal discharge, burning micturition, pruritus vulva, or pain in the lower abdomen (Table 3). Among those who reported symptoms, lower abdominal pain (63%) was most common, followed by burning micturition (58%), itching vulva (39.5%), and abnormal vaginal discharge (34%). Among 222 women who reported vaginal discharge, the discharge was found to be abnormal on examination in 25.7%. One woman had multiple lesions in the vulva and was later confirmed to have a recent syphilis infection. The prevalence of curable STIs was slightly higher, although not significant, among women who reported genitourinary symptoms compared to those who did not (6.3% versus 5.2%, P=0.39). However, curable STIs were significantly more prevalent in women with abnormal vaginal discharge confirmed by gynecological examination than in those reporting no or normal discharge (9.4% versus 5.0%, P=0.005).

### 3.4. Risk Factors for STIs

Trichomoniasis was the only curable STI detected in a sufficient number to explore its risk factors. It was detected more often in the reproductive age group of 15-49 years compared to those > 50 years of age (6.5% versus 2.7%, adjusted OR 2.42, 95% CI 1.18-4.95) (Table 4) and among women from high compared to low cast (10.3% versus 4.7%, adjusted OR 2.25, 95% CI 1.21-4.16). The prevalence was lower among women with husbands with multiple marriages compared to women whose husbands had married only once (1.3% versus 5.9%, adjusted OR 0.20, 95% CI 0.04-0.85). Trichomoniasis was significantly associated with formal education of the woman and also with husband living abroad (OR 2.08 and 2.05, respectively), but not after multivariable adjustment.

## 4. Discussion

We have previously reported a HPV prevalence of 14.3% among women in rural Nepal as well as risk factors for such infection [14]. HPV infection is one of the most common STIs globally [2] and was also the most common STI in this population. In the current study, we examined the prevalence of other STIs and their risk factors in the same population comprising married, nonpregnant women. We observed a low prevalence of curable STIs compared to the most recent global estimates by the WHO for the Southeast Asia region [1], except for trichomoniasis.

The prevalence of trichomoniasis of 5.4% among the women in this study was considerably higher than the global estimate of 1.8% for this region [1]. The prevalence of* C. trachomatis *infection was similar to a population-based study of 1014 postpartum women in a rural district of south eastern Nepal [9] and to that reported from India (0.4%) [17]. However, it was substantially lower than among women in rural areas in China (2.1%) [18] and that estimated for Southeast Asia (1.8%) [1].

The prevalence of syphilis in the general population in Nepal has not been investigated before. As expected the prevalence of syphilis among the rural women in this study (0.2%) was much lower than in a previous study of high-risk populations in Kathmandu and nearby cities, where 0.7% of female sex workers had active syphilis, and 2.5% a previous infection [5]. The estimated prevalence for women in Southeast Asia is 0.37% [1]. In the current study, one of the three women with syphilis had active disease with syphilitic vulvar lesions and concomitant coinfection with different HPV genotypes.

The prevalence of HBsAg of 0.3% was lower than reported for women in Southeast Asia (1.5-3.5%) [19]. HIV infection and gonorrhea were not detected. This is in contrast to previous studies showing a prevalence of 0.2 to 0.3% of HIV in Nepal [4, 11, 20] and an estimated regional variation of 0.3% to 1.7% for gonorrhea around the world [1].

The reason for the low prevalence of most STIs other than HPV infection and trichomoniasis in this study may be that the majority of women were farmers living in a remote area, that they probably rarely leave their village, and that there is hardly any in-migration. Furthermore, most of the husbands had only been married once, and nonmarital sex is prohibited in the Nepalese culture. However, to what degree there is consistency between cultural restrictions and sexual practice may be questioned, since nearly one in ten girls of 12-18 years of age reported to have had sexual experience in a nationwide study from both urban and rural areas [21].

Vaginal discharge was the most frequently reported symptom from the urogenital tract. A diagnosis of curable STIs, mostly trichomoniasis, was associated with vaginal discharge confirmed as abnormal by gynecological examination, but not with self-reported vaginal discharge. Among women who reported vaginal discharge or were confirmed to have abnormal vaginal discharge at gynecological examination, only 6.8% and 9.4%, respectively, were diagnosed with a curable STI. Only 6.3% of those who reported genitourethral symptoms were diagnosed with a curable STI, and half of the women with a curable STI did not report any genitourinary symptoms. Thus, a substantial proportion of STIs occurred in asymptomatic women, who will not be targeted by a syndrome-based approach. The majority of women with* T. vaginalis* infection are asymptomatic [22], and* T. vaginalis* infection has been associated with reproductive morbidity and may facilitate transmission of HIV [23]. This underscores the need for STI screening among asymptomatic women. The fact that no STIs were detected in the majority of the women in this study illustrates the risk of overtreatment if current national guidelines of a syndrome-based approach for STI management are followed. A gynecological examination of women with symptoms would improve the diagnostic accuracy to some extent, but for substantial improvement in the diagnosis of STIs, affordable high quality laboratory diagnostic tests would be needed.

Identification of risk factors for STIs is of major significance for planning of preventive strategies. We and others have previously shown that having multiple partners is associated with HPV infection [1, 14]. In contrast, having a husband with multiple marriages was associated with particularly low prevalence of trichomoniasis in the current study. However, this biologically implausible finding was based on a low number of trichomoniasis cases and may be a chance finding.

In line with previous studies, reproductive age was predictive of trichomoniasis [24]. High cast/ethnicity was also identified as a risk factor for trichomoniasis. This may be attributed to higher socioeconomic class and mobility. We observed a higher prevalence of HPV among smokers [14], which complies with previous studies [25]. An association with smoking has also been shown for other STIs [26]. Due to low prevalence of the other STIs, their risk factors could not be disclosed. However, it is plausible that similar risk factors exist as for HPV and trichomoniasis.

This is the largest study so far conducted on STIs in Nepal. A limitation of the study is that unmarried and pregnant women were not included. This was done to comply with local tradition according to which these two groups of women should not undergo a gynecological examination. The study population was recruited from a limited geographical area and may therefore not be representative for the whole country. However, the majority of the female population in Nepal live in rural areas and belong to a low socioeconomic class similar to the majority of women in this study. An average attendance rate of 62% is acceptable. With the exception of 250 women who were pregnant, data are lacking on the women who did not attend. One likely cause of nonattendance for some of these women would be long walking distance from their home to the one-day screening site. The comparative analysis of urogenital symptoms against laboratory-confirmed STI pathogens was limited by lack of data for yeast infection and bacterial vaginosis, both of which are common causes of genitourinary symptom.

## 5. Conclusion

In this population-based study among married nonpregnant women in rural Nepal, we found a low prevalence of curable STIs compared to regional estimates for Southeast Asia, except for trichomoniasis, which was more prevalent. In the same population, we have previously shown a similar prevalence of HPV infection as estimates for the Southeast Asia region. Vaginal discharge confirmed as abnormal by gynecological examination, but not self-reported discharge, was associated with laboratory diagnosis of a curable STI. Risk factors for trichomoniasis were reproductive age and high cast/ethnicity. A gynecological examination of women with symptoms would improve the diagnostic accuracy to some extent, but for substantial improvement in the diagnosis of STIs, affordable high quality laboratory diagnostic tests are needed. Since STIs are often asymptomatic, screening for asymptomatic persons should also be considered. Our data are important for planning of health services and infection control measures, as well as for diagnosis and treatment of STIs.

## Figures and Tables

**Table 1:** Characteristics of participants in a population-based study of sexually transmitted infections (STIs) among women from rural Nepal (N=1428).

Characteristics	n	%
Age (years)		
15-19	27	1,9
20-29	217	15.2
30-39	370	25.9
40-49	368	25.8
≥ 50	446	31.2
Cast/Ethnicity		
Advantaged high cast	159	11.1
Adhivasi/Janajati	1197	83.8
Disadvantaged low cast	72	5.0
Educational level		
No formal education	1213	84.9
Formal education	215	15.1
Marital status		
Married	1313	91.9
Widow	110	7.7
Divorced	5	0.4
Age when married (years)		
≤15	353	24.7
16-18	485	34.0
≥19	590	41.3
How many times married?		
Once	1378	96.5
More than once	50	3.5
Numbers of births		
0	50	3.5
1-2	409	28.6
3-4	589	41.2
≥5	380	26.6
Husband's residence (N=1313)_ _^a^		
Kavre	1039	79.1
Outside Kavre in Nepal	161	12.3
Abroad	113	8.6
Educational status of husband (N=1312)_ _^a^		
No formal education	715	54.5
Formal education	597	45.5
How many times has husband been married?		
Once	1266	88.7
More than once	162	11.3

^a^Among women whose husbands were alive and information was available.

**Table 2:** Prevalence of sexually transmitted infections (STIs) among women participating in a population-based study in rural Nepal.

Type of STI	Number of samples tested	STI prevalence		
		n	%	95% CI
Curable STIs_ _^a^	1428	82	5.7	4.7-7.1
Trichomoniasis	1306	70	5.4	4.3-6.7
*Chlamydia trachomatis *infection	1329	11	0.8	0.5-1.5
Gonorrhoea	1329	0	0	
Syphilis	1362	3	0.2	0.1-0.6
Viral STIs				
Hepatitis B virus infection	1360	4	0.3	0.1-0.8
Human immunodeficiency virus infection	1360	0	0	
Human papillomavirus infection_ _^b^	1289	186	14.4	12.6-16.5
Any STI_ _^c^	1428	250	17.5	15.6-19.6

^a^Classified as curable STIs by the WHO.

^b^Data from the same population, Shakya S et al. Prevalence of human papillomavirus infection among women in rural Nepal. Acta Obstet Gynecol Scand 2017; 96:29-38.

^c^Presence of any of the STIs listed above.

**Table 3:** Genitourinary symptoms and clinical finding on speculum examination in relation to presence of curable sexually transmitted infections (STIs) in a population-based study among women from rural Nepal.

Genitourinary symptoms and signs	Number of samples tested	Curable STI_ _^a^			
		n	%	95% CI	P- value
Symptoms (N=1428)					
At least one symptom					
Yes	648	41	6.3	4.4-8.2	0.39
No	780	41	5.3	3.7-6.8	
Abnormal discharge					
Yes	222	15	6.8	3.4-10.1	0.48
No	1206	67	5.6	4.3-6.9	
Burning micturition					
Yes	376	26	6.9	4.3-9.5	0.25
No	1052	56	5.3	4.0-6.7	
Itching					
Yes	256	19	7.4	4.2-10.7	0.20
No	1172	63	5.4	4.1-6.7	
Pain lower abdomen					
Yes	408	28	6.9	4.4-9.3	0.25
No	1020	54	5.3	3.9-6.7	
Signs (N=1406)					
Abnormal discharge					
Yes	278	26	9.4	5.9-12.8	0.005
No	1128	56	5.0	3.7-6.2	
Genital ulcer					
Yes	1	1	100		NA
No	1405	81	5.8	4.5-7.0	

^a^Infection with one or more of the STI pathogens *Trichomonas vaginalis, Neisseria gonorrhoeae, Chlamydia trachomatis,* and *Treponema pallidum*. NA: not analysed

**Table 4:** Characteristics of women with *T. vaginalis* infection in a population-based study from rural Nepal (n=70).

	Prevalence			Odds ratios with 95% CI					
				Unadjusted		Age-adjusted		Adjusted_ _^a^	
	n	%	95% CI	OR	95%CI	OR	95%CI	OR	95%CI
Age (years)									
15-49 (n=929)	60	6.5	5.1-8.2	2.53	1.28-5.00	2.53	1.28-5.00	2.42	1.18-4.95
≥ 50 (n=377)	10	2.7	1.4-4.8	1.00	Reference	1.00	Reference	1.00	Reference
Cast/Ethnicity									
Advantaged high cast (n=146)	15	10.3	6.3-16.3	2.30	1.26-4.19	2.42	1.32-4.42	2.25	1.21-4.16
Adhivasi/Janajati/disadvantaged low cast (n=1160)	55	4.7	3.7-6.1	1.00	Reference	1.00	Reference	1.00	Reference
Marital status									
Married (n=1206)	66	5.5	4.3-6.9	1.00	Reference	1.00	Reference	1.00	Reference
Widow/divorced (n=100)	4	4.0	1.6-9.8	0.72	0.26-2.02	1.23	0.41-3.72	1.45	0.47-4.43
Participant's educational status									
No formal education (n=1099)	51	4.6	3.5-6.1	1.00	Reference	1.00	Reference	1.00	Reference
Formal education (n=207)	19	9.2	6.0-13.9	2.08	1.20-3.60	1.68	0.96-2.97	1.65	0.92-2.96
Husband's educational status (N=1205)_ _^b^									
No formal education (n=642)	30	4.7	3.3-6.6	1.00	Reference	1.00	Reference	1.00	Reference
Formal education (n=563)	35	6.2	4.5-8.5	1.35	0.82-2.23	1.10	0.66-1.84	0.89	0.50-1.59
Age at first marriage (years)									
≥ 19 (n=549)	25	4.6	3.1-6.6	1.00	Reference	1.00	Reference	1.00	Reference
16-18 (n=445)	25	5.6	3.8-8.2	1.25	0.71-2.20	1.22	0.69-2.16	1.26	0.71-2.24
≤ 15 (n=312)	20	6.4	4.2-9.7	1.44	0.78-2.63	1.67	0.90-3.08	1.70	0.90-3.21
Numbers of births									
0 (n=44)	3	6.8	2.3-18.2	1.40	0.39-4.98	1.02	0.28-3.68	0.96	0.24-3.84
1-2 (n=386)	23	6.0	4.0-8.8	1.21	0.64-2.31	0.85	0.43-1.67	0.72	0.34-1.50
3-4 (n=534)	27	5.1	3.5-7.3	1.02	0.55-1.90	0.80	0.42-1.52	0.74	0.38-1.42
≥5 (n=342)	17	5.0	3.1-7.8	1.00	Reference	1.00	Reference	1.00	Reference
How many times has participant been married?									
Once (n=1261)	67	5.3	4.2-6.7	1.00	Reference	1.00	Reference	1.00	Reference
More than once (n=45)	3	6.7	2.3-17.9	1.27	0.38-4.21	1.48	0.44-4.96	3.01	0.82-1.03
How many times has husband been married?									
Once (n=1156)	68	5.9	4.7-7.4	1.00	Reference	1.00	Reference	1.00	Reference
More than once (n=150)	2	1.3	0.4-4.7	0.22	0.05-0.89	0.24	0.06-0.98	0.20	0.04-0.85
Husband's residence (n=1206)_ _^a^									
Kavre (n=944)	49	5.2	3.9-6.8	1.00	Reference	1.00	Reference	1.00	Reference
Abroad (n=109)	11	10.1	5.7-17.2	2.05	1.03-4.07	1.69	0.84-3.38	1.67	0.82-3.42
Outside Kavre in Nepal (n=153)	6	3.9	1.8-8.3	0.75	0.31-1.77	0.65	0.27-1.54	0.69	0.29-1.67

^a^Adjusted for age, cast/ethnicity, participant's educational status, age at marriage, and participant's and husband's number of marriages.

^b^Among women whose husbands were alive and information was available.

## Data Availability

The data used to support the findings of this study are available from the corresponding author upon request.

## Supplementary Materials

Supplementary Materials Methods. PCR analysis of* C. trachomatis*,* N. gonorrhoeae, *and* T. vaginalis* and human papillomavirus.
