# Supplementary material for: Prevalence of Sexually Transmitted Infections among Married Women in Rural Nepal
Source: Infect Dis Obstet Gynecol. 2018 Aug 26;2018:4980396. doi: 10.1155/2018/4980396 (PMC6129351; doi:10.1155/2018/4980396)
Supplement: Supplementary Materials — Methods. PCR analysis of C. trachomatis, N. gonorrhoeae, and T. vaginalis and human papillomavirus. [file 4980396.f1.docx]

**Supplementary materials**

**Methods**

PCR analysis of *C. trachomatis*, *N. gonorrhoeae* and *T. vaginalis* and human papillomvirus

Extracted DNA was examined for *N. gonorrhoeae* by an in-house real-time TaqMan PCR for the pseudogene porA with primers and probe as described by Hjelmevoll et al. [1]. The PCR reaction was performed in a final volume of 20 µl, including 15 µl Perfecta qPCR SuperMix with UNG (Quanta Biosciences, Inc., Gaithersburg, MD, USA) with 0.5 µl (300 nM) of each primer, 0.5 µl (200 nM) probe, 3.5 µl PCR-grade water, and 5 µl DNA template. The PCR reaction was run for 5 min at 45°C and 3 min at 95°C, then 40 cycles of 10 s at 95°C and 30 s at 60°C, on a CFX96 Real-Time PCR Detection System (Bio-Rad, CA, USA). *N. gonorrhoeae* strain F-18 (ATCC 49226) was used as positive control.

PCR for *C. trachomatis* was done with primers described by Jaton et al.[2]. The amplification reaction was done in a final volume of 20 µl, including 10 µl SsoFast EvaGreen master mix (Bio-Rad, Hercules, CA, USA), 2 µl (500 nM) of each primer, 1 µl PCR-grade water and 5 µl DNA template, on a CFX96 Real-Time PCR Detection System with the following cycling conditions: 2 min at 90 °C followed by 40 cycles of 2s at 90 °C and 5s at 55 °C. Analysis of amplification products was done by melt point analysis. Lymphogranuloma venereum (LGV II) strain 434 (ATCC VR-902B) was used as positive control.

Molecular detection of *T. vaginalis* was done as singleplex real-time TaqMan PCR using two different primer and probe sets described by Schirm et al. [3]. The PCR reaction was performed in a final volume of 20 µl, including 15 µl Perfecta qPCR SuperMix with UNG (Quanta Biosciences) with 2 µl (500 nM) of each primer, 2 µl (250 nM) probe and 1 µl PCR-grade water, and 5 µl DNA template. The PCR reaction was run for 10 min at 95°C, then 40 cycles of 15 s at 95°C and 60 s at 60°C, on a CFX96 Real-Time PCR Detection System. All samples were first analysed with the most sensitive PCR (L23861 target). Samples with fluorescence signal close to the detection limits were retested by the alternative primer and probe set (L05468 target). These samples were classified as positive only if the alternative PCR was positive. Trichomonas vaginalis DNA control MBC079 (Vircell, Granada, Spain) was used as positive control.

HPV DNA detection was done using the AnyplexTMII HPV28 detection real-time multiplex PCR assay (Seegene Inc., Seoul, Korea) for detection and genotyping of 28 different HPV types (HPV6, 11, 26, 16, 18, 31, 33, 35, 39, 40, 42, 43, 44, 45, 51, 52, 53, 54, 56, 58, 59,61, 66, 68, 69, 70, 73 and 82) as described previously [4]. As endogenous internal control amplification of the β-globin gene was included to verify purity and sufficient amount of extracted human DNA, and efficiency of the amplification reaction in each sample. External positive controls for each HPV subtype were amplified in separate wells.

**References**

1. Hjelmevoll SO, Olsen ME, Sollid JUE, Haaheim H, Unemo M, Skogen V. A fast real-time polymerase chain reaction method for sensitive and specific detection of the Neisseria gonorrhoeae porA pseudogene. The Journal of Molecular Diagnostics. 2006;8(5):574-81.

2. Jaton K, Bille J, Greub G. A novel real-time PCR to detect Chlamydia trachomatis in first-void urine or genital swabs. Journal of medical microbiology. 2006;55(12):1667-74.

3. Schirm J, Bos PA, Roozeboom-Roelfsema IK, Luijt DS, Möller LV. Trichomonas vaginalis detection using real-time TaqMan PCR. Journal of microbiological methods. 2007;68(2):243-7.

4. Shakya S, Syversen U, Åsvold BO, Bofin AM, Aune G, Nordbø SA, et al. Prevalence of human papillomavirus infection among women in rural Nepal. Acta Obstet Gynecol Scand. 2017; 96:29-38.
